# Microglial MT1 activation inhibits LPS‐induced neuroinflammation via regulation of metabolic reprogramming

**DOI:** 10.1111/acel.13375

**Published:** 2021-05-08

**Authors:** Chao Gu, Fen Wang, Yu‐Ting Zhang, Shi‐Zhuang Wei, Jun‐Yi Liu, Hong‐Yang Sun, Guang‐Hui Wang, Chun‐Feng Liu

**Affiliations:** ^1^ Department of Neurology Suzhou Clinical Research Center of Neurological Disease The Second Affiliated Hospital of Soochow University Suzhou China; ^2^ Jiangsu Key Laboratory of Neuropsychiatric Diseases Institute of Neuroscience Soochow University Suzhou China; ^3^ Department of Pharmacy Children’s Hospital of Soochow University Suzhou China; ^4^ Department of Neurology Juntendo University Graduate School of Medicine Tokyo Japan; ^5^ Laboratory of Molecular Neuropathology Jiangsu Key Laboratory of Translational Research and Therapy for Neuropsychiatric Disorders & Department of Pharmacology College of Pharmaceutical Sciences Soochow University Suzhou China

**Keywords:** DA neurons, microglia, MT1, neuroinflammation, Parkinson’s disease, PDHA1

## Abstract

Parkinson’s disease (PD) is one of the most common neurodegenerative diseases. Although its pathogenesis remains unclear, a number of studies indicate that microglia‐mediated neuroinflammation makes a great contribution to the pathogenesis of PD. Melatonin receptor 1 (MT1) is widely expressed in glia cells and neurons in substantia nigra (SN). Neuronal MT1 is a neuroprotective factor, but it remains largely unknown whether dysfunction of microglial MT1 is involved in the PD pathogenesis. Here, we found that MT1 was reduced in microglia of SN in 1‐methyl‐4‐phenyl‐1,2,3,6‐tetrahydropyridine (MPTP)‐induced PD mouse model. Microglial MT1 activation dramatically inhibited lipopolysaccharide (LPS)‐induced neuroinflammation, whereas loss of microglial MT1 aggravated it. Metabolic reprogramming of microglia was found to contribute to the anti‐inflammatory effects of MT1 activation. LPS‐induced excessive aerobic glycolysis and impaired oxidative phosphorylation (OXPHOS) could be reversed by microglial MT1 activation. MT1 positively regulated pyruvate dehydrogenase alpha 1 (PDHA1) expression to enhance OXPHOS and suppress aerobic glycolysis. Furthermore, in LPS‐treated microglia, MT1 activation decreased the toxicity of conditioned media to the dopaminergic (DA) cell line MES23.5. Most importantly, the anti‐inflammatory effects of MT1 activation were observed in LPS‐stimulated mouse model. In general, our study demonstrates that MT1 activation inhibits LPS‐induced microglial activation through regulating its metabolic reprogramming, which provides a mechanistic insight for microglial MT1 in anti‐inflammation.

## Graphical abstract

A schematic diagram shows the involvement of metabolic reprogramming in MT1 activation‐mediated inhibition of LPS‐induced microglial activation. Once microglia suffered LPS insults, microglia would transfer into over‐activated state, accompanied by converting their metabolic status from OXPHOS to aerobic glycolysis. However, microglial MT1 activation could promote PDHA1 expression, leading to reverse LPS‐mediated microglial metabolic reprogramming, thereby, suppressing microglial activation.

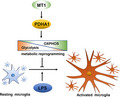

## 1 INTRODUCTION

Neuroinflammation plays an important role in the pathogenesis of many neurodegenerative diseases, including PD (Glass et al., 2010; De Virgilio et al., 2016). Microglia, as critical brain‐resident innate immune cells, play vital roles in brain immune surveillance (Hefendehl et al., 2014; Wolf et al., 2017). However, in face of inflammatory insults, microglia become activated, which release large quantities of pro‐inflammatory cytokines such as tumor necrosis factor alpha (TNF‐α), interleukin‐6 (IL‐6), and interleukin‐1 beta (IL‐1β), which damage neighboring neurons (Colonna & Butovsky, 2017; Glass et al., 2010). DA neurons are sensitive to those pro‐inflammatory mediators (Binukumar et al., 2011). Moreover, activated microglia and pro‐inflammatory cytokines in SN are detected in postmortem PD brains (Hirsch & Hunot, 2009), suggesting a central role of neuroinflammation in the pathogenesis of PD. Moreover, anti‐inflammatory drugs are protective in PD animal models (Gu et al., 2018; Yu et al., 2016). Therefore, inhibiting microglia‐mediated neuroinflammation might ameliorate PD procession.

Although the underlying mechanisms of microglia activation remains ambiguous, recent studies indicate that metabolic reprogramming have shed new light on inflammation (Kelly & O'Neill, 2015). Homeostatic macrophages generate amounts of adenosine triphosphate (ATP) through mitochondrial oxidative phosphorylation (OXPHOS) (Van den Bossche et al., 2017). Upon inflammatory insults like LPS, macrophages exhibit enhanced aerobic glycolysis (Van den Bossche et al., 2017). Such macrophages generate excessive pro‐inflammatory factors (Minton, 2017). Several studies have showed that restriction of aerobic glycolysis remarkably inhibits pro‐inflammatory factors production in macrophages (Nikbakht et al., 2019; O'Neill, 2014). Microglia and macrophages have similar characteristics, demonstrating that regulation of microglial metabolic reprogramming might be an effective means to mitigate neuroinflammation.

Melatonin is an amine hormone secreted by pineal gland in mammals (Hardeland et al., 2011). Its central physiological functions include circadian rhythm modulation and anti‐oxidation effects (Hardeland et al., 2011; Reiter et al., 2000; Sanchez et al., 2015). The majority of the biological functions of melatonin are mediated via its two known G‐protein coupled receptors (GPCR), namely MT1 (encoded by *Mtnr1a*) and MT2 (*Mtnr1b*) (Zlotos et al., 2014). Both receptors are widely distributed in brain (Liu et al., 2016; Ng et al., 2017). It has been reported that MT1 receptors are reduced in AD patients (Wu et al., 2007). Silence of *Mtnr1a* increases the amyloidogenic processing of amyloid precursor protein (Sulkava et al., 2018) and exacerbates mutant huntingtin‐mediated toxicity (Wang et al., 2011), indicating that MT1 has a protective role in neurons. Interestingly, both MT1 and MT2 dramatically down‐regulated in SN of PD patients (Adi et al., 2010), possibly suggesting their vital roles in the progression of PD. However, it is still largely unknown whether and how microglial MT1 is involved in neuroinflammation.

In this study, we assessed the potential protective role of MT1 activation in inflammatory factor‐induced damage of DA neurons. We found that the non‐selective MT1 agonist Ramelteon remarkably inhibits LPS‐induced microglial activation, while silencing of *Mtnr1a* exacerbates microglia‐mediated neuroinflammation both in vivo and in vitro. Moreover, we found that microglial metabolic reprogramming might participate in the MT1‐mediated control of neuroinflammation.

## 2 RESULTS

### 2.1 *Mtnr1a* deficiency aggravates LPS‐induced production of pro‐inflammatory factors in microglia

**FIGURE 1:**
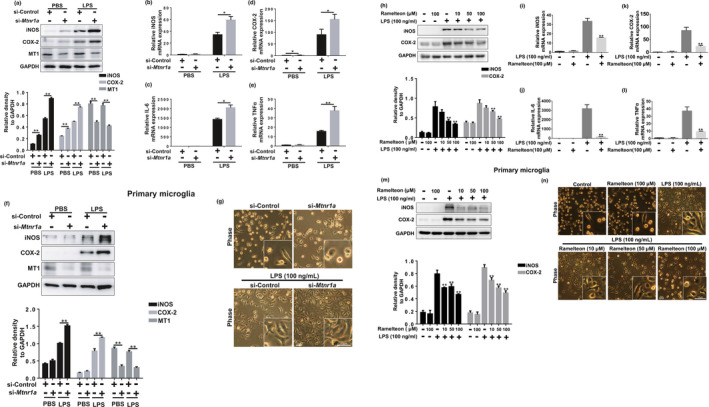
*Mtnr1a* negatively regulates LPS‐induced production of pro‐inflammatory factors in microglia. (a) BV2 cells were transfected with control siRNA or *Mtnr1a* siRNA for 48 hr, then exposed to LPS (100 ng/ml) for 12 hr. After treatments, the protein levels of iNOS, COX‐2, MT1 and GAPDH were determined using immunoblot analyses. The values were presented as the means ± SEM from three independent experiments. **p < 0.01, two‐way ANOVA followed by Sidak’s post‐hoc test. (b–e) BV2 cells were transfected with control siRNA or *Mtnr1a* siRNA for 48 hr, then treated with LPS (100 ng/ml) for 6 hr. After treatments, total RNA were collected to detect the mRNA levels of *iNOS*, *IL*‐*6*, *COX*‐*2* and *TNFα* by qRT‐PCR assays. The values were presented as the means ± SEM from three independent experiments. *p < 0.05, **p < 0.01, two‐way ANOVA followed by Sidak’s post‐hoc test. (f) Primary microglia were treated as (a), the protein levels of iNOS, COX‐2, MT1 and GAPDH were determined using immunoblot analyses. The values were presented as the means ± SEM from three independent experiments. **p < 0.01, two‐way ANOVA followed by Sidak’s post‐hoc test. The morphology of primary microglia were showed in (g). (h) BV2 cells were pretreated with Ramelteon (10 μM, 50 μM, 100 μM) for 12 hr and then exposed to LPS (100 ng/ml) for 12 hr. After treatments, the protein levels of iNOS, COX‐2 and GAPDH were measured using immunoblot analyses. The values were presented as the means ± SEM from three independent experiments. **p < 0.01 vs. the group treated with LPS alone, two‐way ANOVA followed by Sidak’s post‐hoc test. (i–l) BV2 cells were pretreated with Ramelteon (100 μM) for 12 hr and then exposed to LPS (100 ng/ml) for 6 hr. After treatments, total RNA were collected to measure the mRNA levels of *iNOS*, *IL*‐*6*, *COX*‐*2* and *TNFα* by qRT‐PCR assays. The values were presented as the means ± SEM from three independent experiments. **p < 0.01 vs. the group treated with LPS alone, two‐way ANOVA followed by Sidak’s post‐hoc test. (m) Primary microglia were treated as (h), the protein levels of iNOS, COX‐2, MT1 and GAPDH were determined using immunoblot analyses. The values were presented as the means ± SEM from three independent experiments. **p < 0.01, two‐way ANOVA followed by Sidak’s post‐hoc test. The morphology of primary microglia were showed in (n)

It is well established that LPS causes microglia to markedly increase their production and secretion of pro‐inflammatory factors, such as inducible nitric oxide synthase (iNOS), cyclooxygenase‐2 (COX‐2), TNFα, and IL‐6 (Colonna & Butovsky, 2017). We found MT1 was expressed in primary microglia and the murine microglial cell line BV2 (Figure S1a,b). We knocked down *Mtnr1a*, which encodes MT1, in BV2 cells (Figure S2), to assess whether *Mtnr1a* deficiency could potentially affect LPS‐induced microglial activation. We found that LPS significantly promoted the production of iNOS and COX‐2, whereas the transfection of cells with *Mtnr1a* small interfering RNAs (siRNAs) increased the production without affecting the BV2 cell viability (Figure 1a and Figure S3a). In primary microglia, MT1 deficiency also obviously enhanced the expression of LPS‐induced iNOS and COX‐2 (Figure 1f). Activated microglia have properties of amoeboid cells. Primary microglia in which *Mtnr1a* was knocked down, exhibited amoeboid‐like characteristics with an expanded cell body, implying its activation (Figure 1g).

Since we have showed that microglial MT1 deficiency could enhance pro‐inflammatory factors production in protein levels, we carried out an analysis of gene expression. LPS obviously increased the mRNA levels of *iNOS*, *COX*‐*2*, *IL*‐*6*, and *TNF*‐*α*, with an even greater increase in *Mtnr1a*‐knockdown BV2 cells (Figure 1b–e). These data indicated that *Mtnr1a* deficiency in microglia aggravates the production of pro‐inflammatory factors response to LPS.

### 2.2 MT agonist Ramelteon inhibits LPS‐induced production of pro‐inflammatory factors in microglia

Since we found that loss of MT1 in microglia promotes LPS‐induced neuroinflammation, we accessed whether MT1 activation could inhibit LPS‐induced microglial activation. Because no MT1‐specific agonist exists, we used the non‐selective MT agonist Ramelteon, which has been used clinically to treat insomnia (Kuriyama et al., 2014). Ramelteon’s affinity for MT1 is 10‐fold that of MT2 (Zlotos et al., 2014). We found that the abundance of MT1 on microglia was ~3‐fold that of MT2 (Figure S3b), suggesting that Ramelteon indeed acts as an agonist of microglial MT1.

Pretreatment of LPS‐stimulated BV2 cells or primary microglia with Ramelteon significantly suppressed the increase of pro‐inflammatory proteins like iNOS and COX‐2 in a dose‐dependent manner without affecting cell viability (Figure 1h,m, and Figure S3c). Concurrently, the mRNA levels of these pro‐inflammatory factors as well as those of *IL*‐*6* and *TNF*‐*α* were also downregulated (Figure 1i–l). These data indicated that MT1 activation could suppress LPS‐induced microglial activation.

### 2.3 The anti‐inflammatory effect of Ramelteon is specifically dependent on MT1

**FIGURE 2:**
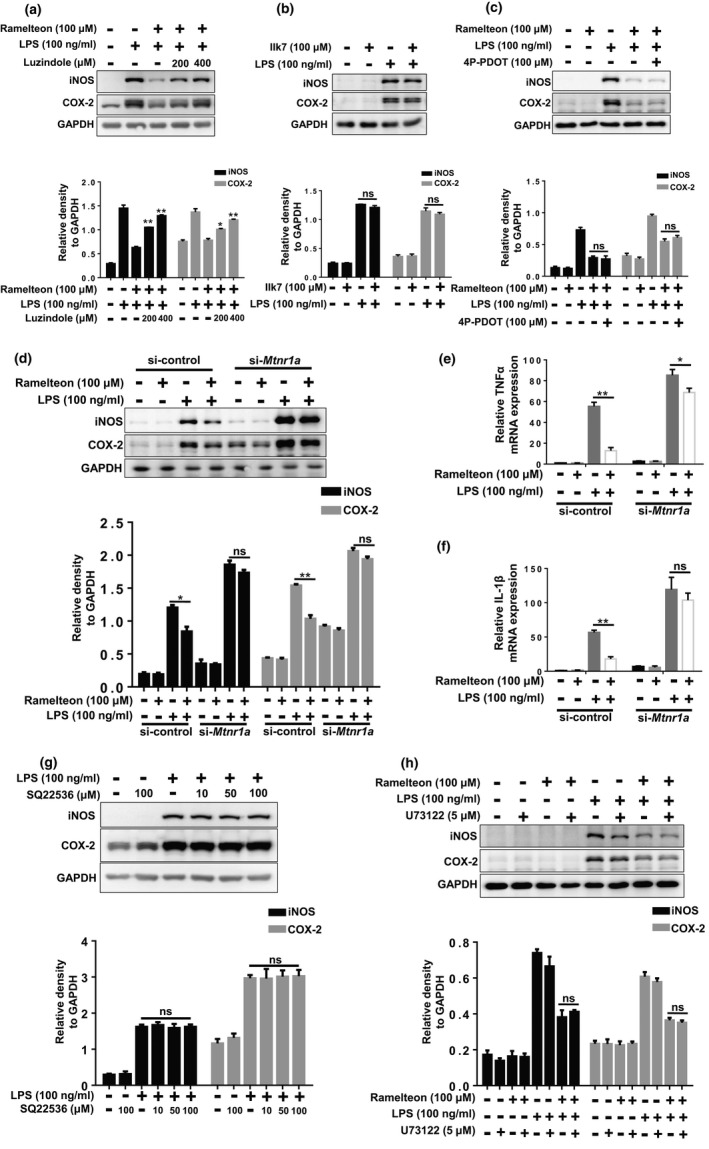
The anti‐inflammatory effect of Ramelteon is specially dependent on MT1. (a) BV2 cells were pretreated with Luzindole (200 μM, 400 μM) for 2 hr, followed by the treatment of Ramelteon for 12 hr (100 μM), then were exposed to LPS (100 ng/ml) for 12 hr. After treatments, the protein levels of iNOS, COX‐2 and GAPDH were measured using immunoblot analyses. The values were presented as the means ± SEM from three independent experiments. *p < 0.05, **p < 0.01 vs. the group treated with LPS +Ramelteon, two‐way ANOVA followed by Sidak’s post‐hoc test. (b) BV2 cells were pretreated with IIK7 (100 μM) for 12 hr, followed by the administration of LPS for 12 hr. Then, the protein levels of iNOS, COX‐2 and GAPDH were measured using immunoblot analyses. The values were presented as the means ± SEM from three independent experiments, ns, no significance, two‐way ANOVA followed by Sidak’s post‐hoc test. (c) BV2 cells were pretreated with 4P‐PDOT (200 μM) for 2 hr, followed by the treatment of Ramelteon for 12 hr (100 μM), then were exposed to LPS (100 ng/ml) for 12 hr. After treatments, the protein levels of iNOS, COX‐2 and GAPDH were measured using immunoblot analyses. The values were presented as the means ± SEM from three independent experiments, ns, no significance, two‐way ANOVA followed by Sidak’s post‐hoc test. (d) BV2 cells were transfected with control siRNA or *Mtnr1a* siRNA for 48 hr, followed by the administration of Ramelteon for 12 hr and then were exposed to LPS (100 ng/ml) for 12 hr. After treatments, the protein levels of iNOS, COX‐2 and GAPDH were measured using immunoblot analyses. The values were presented as the means ± SEM from three independent experiments. *p < 0.05, **p < 0.01, ns, no significance, two‐way ANOVA followed by Sidak’s post‐hoc test. (e, f) BV2 cells were transfected with control siRNA or *Mtnr1a* siRNA for 48 hr, followed by the administration of Ramelteon for 12 hr and then were exposed to LPS (100 ng/ml) for 6 hr. After treatments, total RNA were collected to detect the mRNA levels of *TNFα* and *IL*‐*1β* by qRT‐PCR assays. The values were presented as the means ± SEM from three independent experiments. *p < 0.05, **p < 0.01, ns, no significance, two‐way ANOVA followed by Sidak’s post‐hoc test. (g) BV2 cells were pretreated with SQ22536 (10 μM, 50 μM, 100 μM) for 12 hr and then exposed to LPS (100 ng/ml) for 12 hr. After treatments, the protein levels of iNOS, COX‐2 and GAPDH were measured using immunoblot analyses. The values were presented as the means ± SEM from three independent experiments, ns, no significance, two‐way ANOVA followed by Sidak’s post‐hoc test. (h) BV2 cells were pretreated with U73122 for 2 hr, followed by the administration of Ramelteon for 12 hr, then were exposed to LPS (100 ng/ml) for 12 hr. Thereafter, the protein levels of iNOS, COX‐2 and GAPDH were measured using immunoblot analyses. The values were presented as the means ± SEM from three independent experiments, ns, no significance, two‐way ANOVA followed by Sidak’s post‐hoc test

To exclude any receptor‐independent effects on Ramelteon’s anti‐inflammatory effects, we treated BV2 cells with the MT antagonist Luzindole before Ramelteon treatment. Luzindole inhibited the anti‐inflammatory effects of Ramelteon in a dose‐dependent manner (Figure 2a), suggesting that Ramelteon’s anti‐inflammatory effects are receptor‐dependent.

Because Ramelteon is a non‐selective MT agonist that activates both MT1 and MT2 (Roth et al., 2005), we utilized MT2 specific agonist (IIK7) and antagonist (4P‐PDOT) along with Ramelteon to test whether MT2 is involved in the anti‐inflammatory effects of Ramelteon. In LPS‐treated BV2 cells, neither IIK7 nor MT2 knockdown had any effect on the Ramelteon‐induced anti‐inflammatory response (Figure 2b and Figure S5c), and this was also the case for cells treated with 4P‐PDOT (Figure 2c). In contrast, *Mtnr1a* knockdown in BV2 cells restrained the anti‐inflammatory effects of Ramelteon (Figure 2d–f). These data suggested that MT1 activation is critical in the inhibition of LPS‐induced inflammation.

### 2.4 The anti‐inflammatory effects of MT1 activation are independent of classical GPCR signaling

Since we have showed that MT1 has a significant role in microglial activation, we assessed how microglial MT1 regulates LPS‐induced inflammation. It is well‐known that MT1 is a classical GPCR, which could activate Gαi or Gαq upon ligand binding (Cecon et al., 2018). When Gαi is activated, the activity of adenylate cyclase (AC) is restrained, thereby inhibiting the production of second messenger cyclic adenylate (cAMP) (Cecon et al., 2018). Treatment of BV2 cells with Ramelteon decreased cellular cAMP (Figure S4a), suggesting that this decrease might be involved in the anti‐inflammatory effect of MT1 activation. To test this, we used the compound SQ22536, which downregulates the cellular level of cAMP (Figure S4b), however, LPS‐induced microglial activation was not affected by the pretreatment with SQ22536 (Figure 2g). Thus, the anti‐inflammatory effect of MT1 activation is independent on Gαi signaling.

Next, another MT1‐related G protein Gαq was also tested. The direct downstream signal produced by Gαq is the enhancement of phospholipase C (PLCβ) activity (Cecon et al., 2018), so we examined whether PLCβ activation participates in the anti‐inflammatory effect of MT1 activation. Pretreatment of BV2 cells with the PLCβ inhibitor U73122 prior to administration of Ramelteon and LPS did not affect the anti‐inflammatory effects of Ramelteon (Figure 2h). Thus, Gαq signaling was not involved in the anti‐inflammatory effects of MT1 activation.

### 2.5 Microglial metabolic reprogramming is involved in the anti‐inflammatory effect of MT1 activation

**FIGURE 3:**
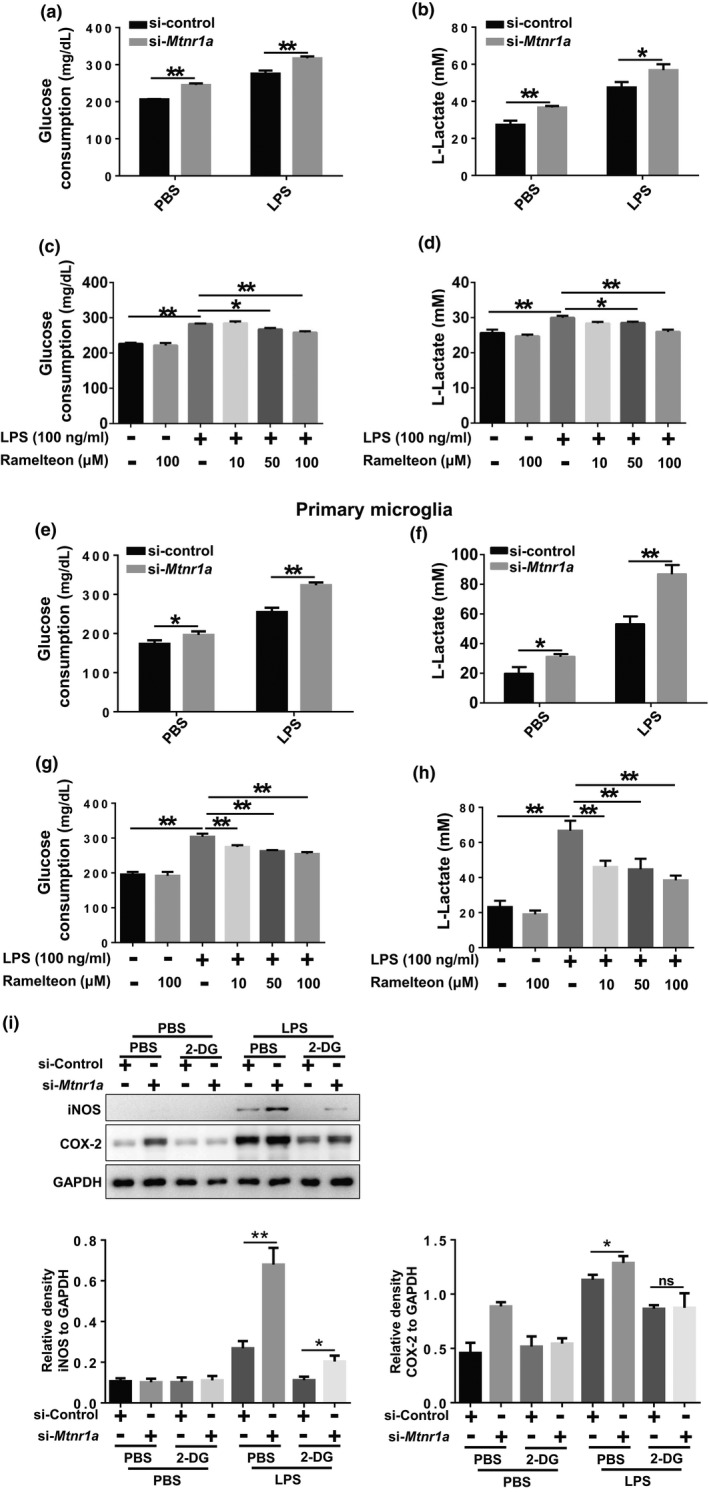
Microglial metabolic reprogramming is involved in the anti‐inflammatory effect of MT1 receptor activation. (a) BV2 cells were transfected with control siRNA or *Mtnr1a* siRNA for 48 hr, then exposed to LPS (100 ng/ml) for 12 hr. After treatments, the culture media were collected and were used to detect the glucose consumption by the QuantiChrom™ Glucose Assay kits. The values were presented as the means ± SEM from three independent experiments. **p < 0.01, two‐way ANOVA followed by Sidak’s post‐hoc test. (b) BV2 cells were treated as described in (a) and then the culture media were collected to determine the lactate production using Lactate Assay kits. The values were presented as the means ± SEM from three independent experiments. *p < 0.05, **p < 0.01, two‐way ANOVA followed by Sidak's post‐hoc test. (c) BV2 cells were pretreated with Ramelteon (10 μM, 50 μM, 100 μM) for 12 hr and then exposed to LPS (100 ng/ml) for 12 hr. After treatments, the culture media were collected and were used to detect the glucose consumption by the QuantiChrom™ Glucose Assay kits. The values were presented as the means ± SEM from three independent experiments. *p < 0.05, **p < 0.01, two‐way ANOVA followed by Sidak’s post‐hoc test. (d) BV2 cells were treated as described in (c) and then the culture media were collected to determine the lactate production using Lactate Assay kits. The values were presented as the means ± SEM from three independent experiments. *p < 0.05, **p < 0.01, two‐way ANOVA followed by Sidak’s post‐hoc test. (e, f) Primary microglia were treated as (a) and (b), after treatments, the culture media were collected and were used to detect the glucose consumption and lactate production by the QuantiChrom™ Glucose Assay kits and Lactate Assay kits. (g‐h) Primary microglia were treated as (c) and (d), after treatments, the culture media were collected and were used to detect the glucose consumption and lactate production by the QuantiChrom™ Glucose Assay kits and Lactate Assay kits. (i) BV2 cells were pretreated with 2‐DG for 2 hr, followed by the knocking down of *Mtnr1a* and a sequential LPS stimulation. Next, the protein levels of iNOS, COX‐2 and GAPDH were measured using immunoblot analyses. Quantitative analyses of panel (i) were shown in panel below. The values were presented as the means ± SEM from three independent experiments. *p < 0.05, **p < 0.01, ns, no significance, two‐way ANOVA followed by Sidak’s post‐hoc test

A growing body of evidence suggests that the pro‐inflammatory factor‐mediated activation of microglia requires metabolic reprogramming (in favor of aerobic glycolysis over oxidative phosphorylation) (Gimeno‐Bayon et al., 2014; Li et al., 2018; Nair et al., 2019; Orihuela et al., 2016). It has been reported that MT1 is an important modulator on insulin secretion in pancreatic β‐cells (Peschke & Muhlbauer, 2010), implying a role of pancreatic β cell‐derived MT1 in regulation of glucose metabolism. We used kits to assay both glucose and lactate to measure glucose consumption and the production of extracellular lactic acid in microglia under different treatments. Consistent with previous studies, LPS promoted glucose consumption and lactic acid production in the cell culture medium (Figure 3a,b,e,f). When *Mtnr1a* was knocked down, glucose consumption and extracellular lactic acid production increased in both BV2 cells (Figure 3a,b) and primary microglia (Figure 3e,f). Conversely, in BV2 cells (Figure 3c,d) or primary microglia (Figure 3g,h) that were pretreated with Ramelteon, glucose consumption and lactic acid production were significantly inhibited. These results indicated that microglial MT1 activation can change its metabolic states from aerobic glycolysis to OXPHOS.

To verify the role of metabolic reprogramming in MT1‐regulated microglial activation, we pretreated BV2 cells with 2‐DG, a glycolytic inhibitor, before knocking down *Mtnr1a*. Interestingly, pretreatment of cells with 2‐DG reversed the increase in LPS‐induced inflammation caused by *Mtnr1a* knockdown (Figure 3i). These data imply that metabolic reprogramming regulation is indeed involved in MT1‐modulated inflammation.

### 2.6 The pyruvate dehydrogenase subunit PDHA1 is involved in MT1‐mediated microglial metabolic reprogramming

**FIGURE 4:**
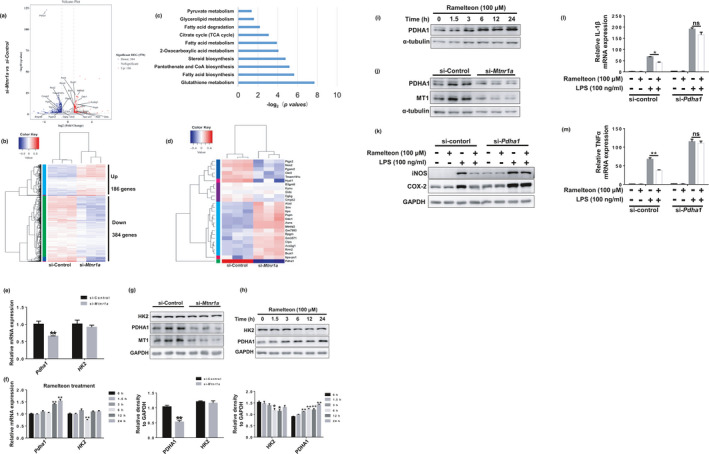
PDHA1 is involved in the regulation of MT1 on metabolic reprogramming. (a) Volcano plot of differentially expressed genes between normal microglia and *Mtntr1a* knocking down microglia. (b) Hierarchical clustering of differentially expressed genes between normal microglia and *Mtntr1a* konckdown microglia. (c) A KEGG (Kyoto Encyclopedia of Genes and Genomes) analysis of the transcriptional program in metabolic pathways induced by microglial *Mtntr1a* knocking down. (d) Heatmap of differentially regulated genes (>2‐folds) in these metabolic pathways including pyruvate metabolism and TCA cycle between si‐Control and si‐*Mtnr1a* group. (e) BV2 cells were transfected with control siRNA or *Mtnr1a* siRNA for 48 hr, then, qRT‐PCR assays were used to detect the mRNA levels of *Pdha1* and *HK2*. The values were presented as the means ± SEM from three independent experiments. **p < 0.01 vs. si‐Control group, two‐way ANOVA followed by Sidak’s post‐hoc test. (f) BV2 cells were treated with Ramelteon (100 μM) in a time‐dependent manner, then the mRNA levels of *Pdha1* and *HK2* were determined by qRT‐PCR assays. The values were presented as the means ± SEM from three independent experiments. **p < 0.01 vs. the group treated with Ramelteon for 0 hr, two‐way ANOVA followed by Sidak’s post‐hoc test. (g) BV2 cells were treated as described in (e), then, immunoblot analyses were used to detect the protein levels of PDHA1 and HK2. The values were presented as the means ± SEM from three independent experiments. **p < 0.01 vs. si‐Control group, two‐way ANOVA followed by Sidak’s post‐hoc test. (h) BV2 cells were treated as described in (f), then, immunoblot analyses were used to detect the protein levels of PDHA1 and HK2. *p < 0.05, **p < 0.01 vs. the group treated with Ramelteon for 0 hr, two‐way ANOVA followed by Sidak’s post‐hoc test. (i) Primary microglia were treated as (h), immunoblot analyses were used to detect the protein levels of PDHA1. (j) Primary microglia were treated as (g), immunoblot analyses were used to detect the protein levels of PDHA1. (k) BV2 cells were transfected with control siRNA or *Pdha1* siRNA for 48 hr, then with a 12‐h Ramelteon treatment, followed by LPS stimulation for 12 hr. The protein levels of iNOS and COX‐2 were detected with immunoblot analyses. (l, m) BV2 cells were transfected with control siRNA or *Pdha1* siRNA for 48 hr, then with a 12‐hr Ramelteon treatment, followed by LPS stimulation for 6 hr. After treatments, total RNA were collected to detect the mRNA levels of *TNFα* and *IL*‐*1β* by qRT‐PCR assays. The values were presented as the means ± SEM from three independent experiments. *p < 0.05, **p < 0.01, ns, no significance, two‐way ANOVA followed by Sidak’s post‐hoc test

As we found that MT1 activation inhibits LPS‐induced microglial activation through regulating metabolic reprogramming, we wonder the potential mechanisms underlying it. We isolated and sequenced total RNA from *Mtnr1a*‐deficient and normal microglia. A total of 186 genes were upregulated and 384 genes were downregulated in *Mtnr1a*‐deficient microglia compared with the normal group (Figure 4a,b). Based on the Kyoto Encyclopedia of Genes and Genomes (KEGG), many of these genes are associated with certain metabolic pathways, including pyruvate metabolism, the citric acid cycle, and fatty acid metabolism (Figure 4a,c). A heatmap revealed genes with significant changes in expression (i.e., >2‐fold) in certain metabolic pathways, and we were amazed to find that *Pdha1* expression was decreased substantially in *Mtnr1a*‐deficient microglia (Figure 4d). *Pdha1* encodes the E1 alpha 1 subunit of pyruvate dehydrogenase (PDH) complex which has a critical role in the citric acid cycle (Tan et al., 2015).

To verify RNA sequencing results, we knocked down *Mtnr1a* or used Ramelteon to treat BV2 cells. At the transcriptional level, *Pdha1* expression decreased significantly in *Mtnr1a*‐deficient BV2 cells (Figure 4e), whereas it increased substantially in Ramelteon‐treated (MT1‐activated) cells. Similarly, MT1 positively regulate PDHA1 expressions in protein levels in both BV2 cells (Figure 4g, h) and primary microglia (Figure 4i, j). To confirm that PDHA1 is involved in regulating MT1‐mediated inhibition of LPS‐induced neuroinflammation, we knocked down *Pdha1* in BV2 cells before Ramelteon treatment. *Pdha1* knockdown nearly eliminated the anti‐inflammatory effects of MT1 activation (Figure 4k–m).

### 2.7 MT1 activation inhibits microglia‐mediated DA neuronal death

**FIGURE 5:**
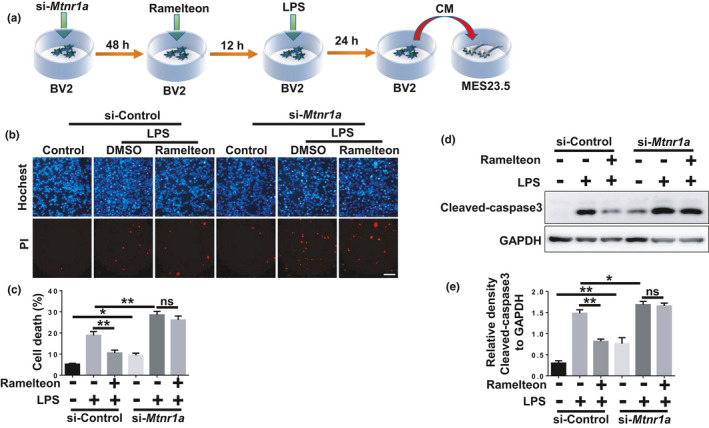
MT1 activation inhibits microglia‐mediated DA neuronal death. (a) A scheme of the conditioned medium assay using the media collected from Ramelteon‐pretreated plus LPS‐stimulated BV2 cells with or without the loss of *Mtnr1a* to culture MES 23.5 cells. (b) BV2 cells were transfected with control siRNA or *Mtnr1a* siRNA for 48 hr, then exposed to LPS (100 ng/ml) for 24 hr with the pretreatment of Ramelteon (100 μM), then the conditioned media from different groups were collected to culture the MES23.5 cells for 24 hr. After treatment, the MES23.5 cells were subjected to Hoechst or PI staining to detect cell death. Scale bar, 10 μm. Quantitative analyses of panel (b) were shown in panel (c). (d) BV2 cells were treated as (b) and subjected to immunoblot analyses to detect the protein level of Cleaved‐caspase3 and GAPDH. Quantitative analyses of panel (d) were shown in panel (e). The values were presented as the means ±SEM from three independent experiments. **p < 0.01, ns, no significance, two‐way ANOVA followed by Sidak’s post‐hoc test

It is well accepted that activated microglia could release a number of pro‐inflammatory factors that damage neighboring neurons (Wolf et al., 2017). As we showed that MT1 activation has remarkable anti‐inflammatory effects, we speculated that MT1 may mediate the neurotoxicity caused by activated microglia. To verify our speculation, we prepared conditioned medium (CM) from Ramelteon‐pretreated, LPS‐stimulated BV2 cells with or without *Mtnr1a* knockdown. Conditioned medium was then collected for culture of the DA cell line MES23.5 (Figure 5a). Thereafter, MES23.5 cells were stained with Hochest and propidium iodide (PI) (Figure 5b,c), and cleaved‐caspase3 was assayed for evaluating cell apoptosis (Figure 5d,e). The neurotoxicity of CM from LPS‐stimulated BV2 cells was obviously increased, whereas cell survival increased markedly when cells were cultured with CM from LPS‐stimulated BV2 cells that were pretreated with Ramelteon. Moreover, CM from *Mtnr1a*‐knockdown BV2 cells increased cell death of MES23.5 compared with that from the control group (Figure 5b–e). However, this protective effect of Ramalteon CM was almost abolished by *Mtnr1a* knockdown in BV2 cells (Figure 5b–e). These data suggested that MT1 activation attenuates the microglia‐mediated death of DA neurons.

### 2.8 **Ramelteon inhibits microglial activation and microglia‐mediated DA neuronal loss in a mouse model of LPS‐induced neuroinflammation**.

**FIGURE 6:**
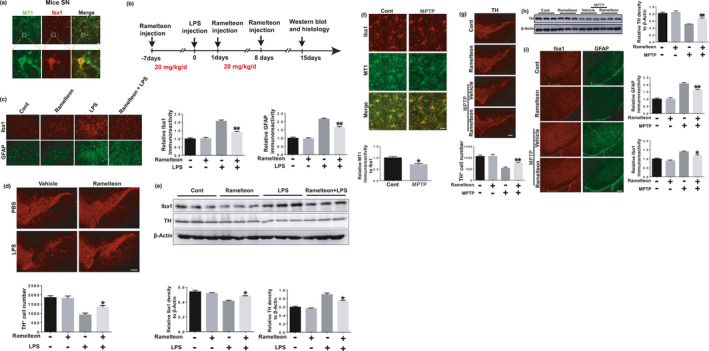
MT1 activation inhibits microglial activation and microglia‐mediated DA neuronal loss in LPS and MPTP‐induced mouse model. (a) The co‐location of MT1 (green) and Iba1 (red) in microglia in mice SN. Scale bar, 50 μm. (b) A schematic diagram showed the animal experimental procedure. (c) Immunohistochemical staining was performed via anti‐Iba1 and anti‐GFAP antibodies. Scale bar, 50 μm. *n* = 5 per group. **p < 0.01 vs. the group treated with LPS alone using two‐way ANOVA followed by Sidak’s post‐hoc test. (d) Immunohistochemical staining was conducted using anti‐TH antibodies. The quantification of TH^+^ cell numbers was shown in below. Scale bar, 50 μm. *n* = 4 per group. *p < 0.05 vs. the group treated with LPS alone, two‐way ANOVA followed by Sidak's post‐hoc test. (e) Mice midbrains were isolated and the proteins were collected to measure the protein levels of Iba1 and TH in different groups using immunoblot analyses. Quantitative analyses of panel (h) were shown in panel below. *n* = 3 per group. *p < 0.05 vs. the group treated with LPS alone, two‐way ANOVA followed by Sidak’s post‐hoc test. (f) The co‐location of MT1 (green) and Iba1 (red) in microglia in mice SN. Scale bar, 10 μm, *n* = 4 per group, the relative fluorescence intensity of MT1 to Iba1 was shown in below. *p < 0.05 vs. the group treated with MPTP using one‐way ANOVA followed by Sidak’s post‐hoc test. (g) Immunohistochemical staining was conducted using anti‐TH antibodies. The quantification of TH^+^ cell numbers was shown in below. Scale bar, 50 μm. *n* = 4 per group. **p < 0.01 vs. the group treated with MPTP alone, two‐way ANOVA followed by Sidak’s post‐hoc test. (h) Mice SN were isolated and the proteins were collected to measure the protein levels of TH in different groups using immunoblot analyses. Quantitative analyses were shown in right. *n* = 3 per group. **p < 0.01 vs. the group treated with MPTP alone, two‐way ANOVA followed by Sidak’s post‐hoc test. (i) Immunohistochemical staining was performed via anti‐Iba1 and anti‐GFAP antibodies and the relative fluorescence intensity of Iba‐1 and GFAP were shown in right. Scale bar, 50 μm. *n* = 3 per group. **p < 0.01 vs. the group treated with MPTP alone using two‐way ANOVA followed by Sidak’s post‐hoc test

Since we found that MT1 activation has dramatic anti‐inflammatory effects *in vitro*, we next tested whether MT1 activation could inhibit microglial activation *in vivo*. Therefore, we used a mouse model of LPS‐induced inflammation via stereotactic injections of LPS into the SN of mice. MT1 was expressed in microglia in mice SN (Figure 6a). Ramelteon was injected intraperitoneally before and after the LPS injections (Figure 6b), with subsequent analysis by immunohistochemistry. LPS treatment substantially increased the levels of both Iba1 (microglia marker) and GFAP (glial acidic fibrillary protein; astrocyte marker) in the SN. However, in Ramelteon‐treated groups, LPS‐induced expressions of Iba1 and GFAP decreased markedly in the SN (Figure 6c). Moreover, LPS‐induced tyrosine hydroxylase (TH^+^) neuronal loss was also substantially attenuated (Figure 6d). To further identify the anti‐inflammatory effects of MT1 activation in vivo, we prepared total‐protein extracts of the mouse midbrain from the various treatment groups. The LPS‐induced increase of Iba1 and decrease of TH expressions in the mouse midbrain were notably reversed when the mice were pretreated with Ramelteon (Figure 6e).

### 2.9 **Ramelteon inhibits DA neuronal loss and microglial activation in MPTP‐induced PD mouse model**.

To further explore the protective effects of MT1 activation on DA neuronal loss *in vivo*, we performed the MPTP‐induced PD model. A seven consecutive days Ramelteon treatment were conducted before and when MPTP intraperitoneal injections (Figure S5a). In the mouse SN, MPTP insults induced microglial MT1 reduction (Figure 6f), which is consistent with the clinical report that MT1 abundance was decreased in SN of PD patients (Adi et al., 2010). Moreover, Ramelteon treatment alleviated MPTP‐induced loss of TH^+^ neurons (Figure 6g) and the decrease of TH expressions in SN (Figure 6h). We compared the expressions of MT1 in cultured primary microglia, astrocyte and neurons, and found that the higher MT1 expression in microglia and neurons (Figure S5b), this might imply that MT1 of DA neurons also act as a role in neuroprotection. It is possible that MPTP could directly cause the death of DA neurons, whereas LPS‐mediated damage to DA neurons may occur through microglia‐mediated inflammation. Injured or dead DA neurons could also make neighboring glia activated, so we assessed glial activation in the SN. The abundance of both Iba1 and GFAP increased in the SN of the MPTP‐treated groups, whereas Ramelteon inhibited this increase (Figure 6i). Therefore, the MT agonist Ramelteon was neuroprotective in MPTP‐induced PD mouse model.

## 3 DISCUSSION

Our present study clarifies the role of microglial MT1 in neuroinflammation. We found that microglial MT1 regulates neuroinflammation via regulating metabolic reprogramming. MT1 activation forces activated microglia to alter their metabolic state from aerobic glycolysis to oxidative phosphorylation, thereby inhibiting microglial activation induced by inflammatory stimuli such as LPS or MPTP, leading to a further protection of DA neurons from pro‐inflammatory damage.

MT1 plays a key role in neurodegenerative diseases (Sulkava et al., 2018; Suofu et al., 2017; Wang et al., 2011). Interestingly, MT1 is highly expressed in SN (Lacoste et al., 2015) but is significantly reduced in SN of PD patients (Adi et al., 2010), suggesting that MT1 dysfunction might be a potential risk for PD. Our present study reveals that MT1 activation inhibits LPS‐induced neuroinflammation, whereas *Mtnr1a* deficiency promotes it both in vivo and in vitro. Most importantly, MT1 activation remarkably inhibits DA neurons damage in MPTP‐induced PD mouse model. Because a selective MT1 agonist does not exist, we used Ramelteon, which activates both MT1 and MT2. In microglia, MT1 abundance is relatively high and is three times that of MT2 (Figures S3b and S5b); moreover, Ramelteon had almost 10‐fold greater affinity for MT1 than MT2 (Kato et al., 2005), implying that the effects of Ramelteon are mediated predominantly by MT1. Even so, we cannot exclude potential contributions of other unknown targets of Ramelteon, and indeed this might explain why there are some inconformity between MT1 knocking down and Ramelteon treatment.

Mechanically, we found PDHA1 is the key factor of MT1‐mediated neuroinflammation. In pancreatic islet B cells, MT1 function is coupled to the canonical G protein–coupled receptor, Gαq, to promote insulin secretion (Peschke & Muhlbauer, 2010), indicating that MT1 is involved in glucose metabolism. Moreover, recent studies have shown that metabolic reprogramming of microglia is important for regulate their activation. Upon activation, the metabolic state of activated microglia changes from oxidative phosphorylation to aerobic glycolysis (Gimeno‐Bayon et al., 2014; Li et al., 2018), suggesting a link between MT1 function and microglial glycolysis. We found that MT1 activation positively regulated PDHA1. *Pdha1* encodes the E1 alpha 1 subunit of PDH complex which acts as a critical role in the citric acid cycle (Tan et al., 2015). Many studies have shown that PDHA1 regulates metabolic reprogramming in macrophages (Semba et al., 2016; Tan et al., 2015). Interestingly, PDHA1 has been shown to be substantially downregulated in the SN of PD patients (Miki et al., 2017).

PDHA1 is a mitochondrial localization factor for nuclear‐encoded proteins, but it remains unclear how MT1 regulates *Pdha1* expression. One study reported that the MT1 also exists on the mitochondrial membrane in neurons (Suofu et al., 2017), suggesting MT1 may directly combined with PDHA1 in microglial mitochondria and regulated its expression. In addition we found that MT1 regulates PDHA1 abundance at both the translational and transcriptional levels. One study showed that peroxisome proliferator‐activated receptor γ coactivator‐α (PGC1α) positively regulates *PDHA1* transcription in human hepatobiliary carcinoma cells (Dan et al., 2018). However, our study shows that MT1 has no significant effect on PGC1α. The mechanisms by which MT1 regulates PDHA1expression needs further exploration.

We believe MT1 might play a vital role in inflammation‐associated neurodegenerative diseases. Certain MT1 agonists, including Agomelatine, Ramelteon, and Tasimelteon that are used clinically for sleep disorders (Kato et al., 2005; Kuriyama et al., 2014; Liu et al., 2016), have the potential to inhibit neuroinflammation, further remitting neuron loss and alleviate motor symptoms in PD or other neurodegenerative diseases like AD. All these agonists are non‐selective, although it may be possible to design MT1‐specific agonists based on analysis of the crystal structure for human MT1 (Stauch et al., 2019). Such MT1‐specific agonists may provide better therapeutic efficacy for sleep disorders while helping prevent neuronal loss. We believe MT1‐specific agonists may have great potential for treating certain neurodegenerative diseases that are commonly accompanied by sleep disorders, for example, PD and AD.

## 4 EXPERIMENTAL PROCEDURES

### 4.1 Animal experiments

Male C57BL/6 mice (age 6–8 months, 20–25 g) were purchased from JIHUI Animal Ltd. The mice were housed on the conditions of 20–26°C, 50%–60% relative humidity and a 12‐hr light and a 12‐hr dark cycle for 2 weeks before experimentation. Mice had free access to water and food. All experiments were performed according to the Regulations of Experimental Animal Administration issued by the Animal Committee of Soochow University. For mouse model of LPS‐induced inflammation, the mice were divided into four groups randomly: (1) vehicle +PBS group; (2) Ramelteon +PBS group; (3) vehicle +LPS group; (4) Ramelteon +LPS group. Each mouse in the group (2) and (4) was administrated with Ramelteon (20 mg/kg per day) for seven consecutive days through intraperitoneal injection, whereas each mouse in the other groups received an equal volume of vehicle. Vehicle contains a final formulation of 3% dimethyl sulfoxide (DMSO), 10% cremophor EL, 87.5% D5 W (5% dextrose in water, pH 7.2). On the day 8, the mice in the group (3) and (4) were treated with LPS (4 μg) to the bilateral SN by stereotaxic injection at AP injection at AP −3.3 mm, ML ±1.2 mm, DV −4.6 mm from bregma. One day after, the mice in group (2) and (4) were treated with another 7‐days Ramelteon intraperitoneal injection. For MPTP‐induced PD mouse model, the mice were divided into four groups randomly: (1) vehicle +saline group; (2) Ramelteon +Saline group; (3) vehicle +MPTP group; (4) Ramelteon +MPTP group. Each mouse in the group (2) and (4) were administrated with Ramelteon at a dose of 20 mg kg^−1^ day^−1^ for consecutive 7 days through intraperitoneal injection, while each mouse in the other groups received an equal volume of vehicle. The mice in the group (3) and (4) were treated with MPTP along with Ramelteon for another 7 days.

### 4.2 Cell culture and drugs treatment

BV2 cells, a murine microglial cell line, were maintained in Dulbecco’s modified Eagle’s medium (DMEM) with 10% heat‐inactivated fetal bovine serum, 100 μg/ml penicillin and 100 μg/ml streptomycin. MES23.5 cells, a dopaminergic cell line, were cultured in DMEM/F12 with Sato’s components containing 5% heat‐inactivated fetal bovine serum including penicillin (100 mg/ml) and streptomycin (100 mg/ml). Primary cultured microglia were acquired and cultured as described before (Gu et al., 2018). Ramelteon, IIK7, 4P‐PDOT, Luzindole, SQ22536, and U73122 were purchased from Selleck technology and dissolved in DMSO. LPS was purchased from Sigma and dissolved in PBS.

### 4.3 Cell viability assay

3‐(4,5‐dimethylthiazol‐2‐yl)‐2,5‐diphenyltetrazolium bromide (MTT) assays were performed to assess the cell viability. BV2 cells were pretreated with varying concentrations of Ramelteon or with siRNAs against *Mtnr1a*, followed by the LPS administration. Thereafter, BV2 cells were incubated with 0.5 mg/ml MTT at 37°C for 2 hr. The reaction was then stopped with 150 μl of DMSO. The absorbance was detected at 570 nm to determine the cell viability.

### 4.4 RNA interference

RNAiMAX (Invitrogen) was used for transfection of BV2 cells or primary microglia with RNA oligonucleotides. The cells were incubated in a mixture of RNAiMAX, Opti‐MEM and RNA oligonucleotides for 15 min at room temperature. After 24 hr, the media were replaced with fresh media. Another 24 hr later, the cells were treated with Ramelteon or 2‐DG. The oligonucleotides that target mouse *Mtnr1a* or *Pdha1* were purchased from GenePharma technology. The sequences were as follows: si‐*Mtnr1a*‐1: sense 5’‐UUAUUAUUGGGUAUUAGUGGA‐3’, antisense 5’‐ CACUAAUACCCAAUAAUAACU‐3’; si‐*Mtnr1a*‐2: sense 5’‐ UUUAUUAAGUUAUUAUUGGGU‐3’, antisense 5’‐CCAAUAAUAACUUAAUAAAGG‐3’; si‐*Mtnr1a*‐3: sense 5’‐ UAUUGUAAUCCUAAAUGUGGG‐3’, antisense 5’‐ CACAUUUAGGAUUACAAUAUA‐3’; si‐*Pdha1*‐1: sense 5’‐ UUCUUAAUCUCAAAUGUAGCA‐3’, antisense 5’‐ CUACAUUUGAGAUUAAGAAAU‐3’; si‐*Pdha1*‐2: sense 5’‐ AUGUUAAAUGUCUAAAUGGAA‐3’, antisense 5’‐ CCAUUUAGACAUUUAACAUAC‐3’; si‐*Pdha1*‐3: sense 5’‐ UAUAAACUAGUGUAAUUACUU‐3’, antisense 5’‐ GUAAUUACACUAGUUUAUAAA‐3’.

### 4.5 RNA extraction, reverse transcription‐PCR and real‐time quantitative RT‐PCR

After different treatments of BV2 cells, the total RNA were obtained using TRIzol Reagent (Invitrogen). Then, we used PrimeScript RT Master Mix (Takara) to prepare cDNA. The real‐time quantitative PCR was performed using SYBR Green PCR Master Mix (Applied Biosystems) and the products were measured using an Applied Biosystems 7500 Real Time‐PCR System (Applied Biosystems). The sequences of PCR primers were as follows: mouse *β*‐*actin*: 5′‐GACCTGACTGACTACCTC‐3′ and 5′‐GACAGCGAGGCCAGGATG‐3. *iNOS*: 5′‐TCCCAGCCTGCCCCTTCAAT‐3′ and 5′‐CGGATCTCTCTCCTCCTGGG‐3′, mouse *COX*‐*2*: 5′‐ACACTCTATCACTGGCACCC‐3′ and 5′‐GAAGGGACACCCCTTCACAT‐3′, mouse *TNF*‐*α*: 5′‐CTCATCAGTTCTATGGCCCAG‐3′ and 5′‐CGCAAAGTCTAAGTACTTGG‐3′, *IL*‐*6*: 5′‐ GTCCTTCAGAGAGATACAGAAACT‐3′ and 5′‐AGCTTATCTGTTAGGAGAGCATTG‐3′, mouse *HK2*: 5′‐TGATCGCCTGCTTATTCACGG‐3′ and 5′‐AACCGCCTAGAAATCTCCAGA‐3′, mouse *Mtnr1a*: 5′‐TGTCAGCGAGCTGCTCAATG‐3′ and 5′‐GGTACACAGACAGGATGACCA‐3′, mouse *Mtnr1b*: 5′‐GAACAGCTCAATCCCTAACTGC‐3′ and 5′‐ACGACTACTGTAGATAGCATGGG‐3′, mouse *Pdha1*: 5′‐GAAATGTGACCTTCATCGGCT‐3′ and 5′‐TGATCCGCCTTTAGCTCCATC‐3′. Relative mRNA expression levels were calculated using the $2^{\Delta \Delta C_{t}}$ method.

### 4.6 Glucose and lactate detection

After different treatments of BV2 cells or primary microlgia, the culture media were collected and the lactate levels in different media were assessed via Lactate Assay kits (BioAssay Systems) according to manufacturer’s instructions. Glucose consumptions in different groups were also determined using QuantiChrom™ Glucose Assay kits (BioAssay Systems) according to manufacturer’s instructions.

### 4.7 Immunoblot analysis and antibodies

After treatments, BV2 cells and primary microlglia or isolated tissues that had been homogenized by PRO200 homogenizer were lysed in 1 × SDS lysis buffer (150 mmol/L NaCl, 25 mmol/L Tris‐HCl, pH 7.6, 1% sodium deoxycholate, and 1% NP‐40) with a phosphatase inhibitor and a protease inhibitor (Roche). About 20 μg of cell lysate was isolated with SDS‐PAGE and then transferred onto a PVDF membrane (Millipore). Immunoblot analysis were performed with the following primary antibodies: anti‐iNOS, anti‐COX‐2, anti‐α‐Tubulin, anti‐β‐Actin (Abcam); anti‐MT1 (Santa Cruz Biotechnology); anti‐GAPDH antibodies (Millipore); anti‐HK2, anti‐PDHA1, anti‐Cleaved‐caspase‐3 (Cell Signaling Technology); anti‐Iba1 (Wako Chemicals); anti‐TH (Millipore). The secondary antibodies, sheep anti‐rabbit or anti‐mouse IgG‐HRP, were purchased from Thermo Fisher Scientific. The proteins were visualized using ECL detection kits (Thermo Fisher Scientific).

### 4.8 Immunofluorescence Staining

BV2 cells or Primary microglia were fixed with 4% paraformaldehyde in PBS, followed by permeabilizing with 0.25% Triton X‐100 in PBS for 5 min at room temperature. Then, the cells were washed with PBS for three times and blocked with 0.5% fetal bovine serum. Thereafter, the cells were incubated with anti‐Iba1 (Wako Chemicals) or anti‐MT1 (Santa Cruz Biotechnology) for 6 hr at room temperature, followed by an incubation with rhodamine‐conjugated (red) ‐or FITC (green)‐conjugated secondary antibody for 2 hr. Subsequently, the cells were stained with DAPI (Sigma) for 5 min. Finally, the cells were observed using an inverted IX71 microscope system (Olympus).

### 4.9 Immunohistochemistry

After the treatments of Ramelteon and LPS or MPTP described above, the mice were perfused with 0.9% saline followed by 4% paraformaldehyde in 0.1 M PBS (pH 7.4). The mice brains were then removed and post‐fixed in the same fixation agent overnight at 4°C, followed by a treatment with 30% sucrose at 4°C with another night. Serial 20 μM‐thick mouse midbrain slices were cut with a freezing microtome. Immunohistochemical staining was conducted with anti‐MT1 antibody, anti‐Iba1antibody (Wako Chemicals), anti‐GFAP, and anti‐TH antibodies (Millipore) against six slices per mouse (120 μm interval). After incubation with primary antibodies at room temperature for 6 hr, the slices were incubated with rhodamine (red)‐or FITC (green)‐conjugated secondary antibody (Invitrogen) for 2 hr. Thereafter, the slices were stained with DAPI for 5 min and observed using an inverted IX71 microscope system (Olympus). The number of TH^+^ neurons and the fluorescence intensity of MT1, Iba1, and GFAP were counted using Image J (National Institute of Health).

### 4.10 RNA sequencing and bioinformatics analyses

Total RNA of each sample was extracted using TRIzol Reagent (Invitrogen)/RNeasy Mini Kit (Qiagen)/other kits. Total RNA of each sample was quantified and qualified by Agilent 2100 Bioanalyzer (Agilent Technologies), NanoDrop (Thermo Fisher Scientific Inc.) and 1% agrosegel. 1 μg total RNA with RIN value above 7 was used for following library preparation. Next generation sequencing library preparations were constructed according to the manufacturer’s protocol (NEBNext^®^ Ultra™ RNA Library Prep Kit for Illumina^®^). Then libraries with different indices were multiplexed and loaded on an Illumina HiSeq instrument according to manufacturer’s instructions (Illumina). Sequencing was carried out using a 2 × 150 bp paired‐end (PE) configuration; image analysis and base calling were conducted by the HiSeq Control Software (HCS) + OLB + GAPipeline‐1.6 (Illumina) on the HiSeq instrument. The sequences were processed and analyzed by GENEWIZ.

Firstly, reference genome sequences and gene model annotation files of relative species were downloaded from genome website. Secondly, Hisat2 (v2.0.1) was used to index reference genome sequence. Finally, clean data were aligned to reference genome via software Hisat2 (v2.0.1). Differential expression analysis used the DESeq Bioconductor package, a model based on the negative binomial distribution. After adjusted by Benjamini and Hochberg’s approach for controlling the false discovery rate, *p*‐value of genes were set <0.05 to detect differential expressed ones. KEGG is a collection of databases dealing with genomes, biological pathways, diseases, drugs, and chemical substances (http://en.wikipedia.org/wiki/KEGG). We used scripts in house to enrich significant differential expression gene in KEGG pathways.

### 4.11 Statistical analysis

Two‐way ANOVA followed by Sidak’s post‐hoc test was performed to analyze differences in different treatments. All analyses were performed using GraphPad Prism version 7.00 (GraphPad Software). *P* values <0.05 was considered as significant difference. All values are displayed as the mean ± SEM. *p < 0.05, **p < 0.01.

## CONFLICT OF INTEREST

The authors declare no competing financial interests.

## AUTHOR CONTRIBUTIONS

C.‐F.L. and C.G. designed the study. C.G. drafted the manuscript. C.G., Y.‐T.Z. and S.‐Z.W. performed research. C.‐F.L. and G.‐H.W. revised the manuscript. J.‐Y.L., H.‐Y.S. and F.W. discussed the data. C.G. analyzed the data. F.W. contributed critical reagents.

## Supporting information

Supplementary Material

## Data Availability

Our RNA‐seq RAW data are available in: https://eur03.safelinks.protection.outlook.com/?url=https%3A%2F%2Fwww.ncbi.nlm.nih.gov%2Fsra%2F%3Fterm%3DPRJNA721745&data=04%7C01%7Cisabell.mayr%40kcl.ac.uk%7Cb5b91912f3c44a40df5408d8ffb60921%7C8370cf1416f34c16b83c724071654356%7C0%7C0%7C637540504596379386%7CUnknown%7CTWFpbGZsb3d8eyJWIjoiMC4wLjAwMDAiLCJQIjoiV2luMzIiLCJBTiI6Ik1haWwiLCJXVCI6Mn0%3D%7C1000&sdata=7aPugNbmvwVzd0veL%2F7CT8JkfPzRffk4%2FyZgfthK0mc%3D&reserved=0.

## References

[acel13375-bib-0001] Adi, N. , Mash, D. C. , Ali, Y. , Singer, C. , Shehadeh, L. , & Papapetropoulos, S. (2010). Melatonin MT1 and MT2 receptor expression in Parkinson's disease. Medical Science Monitor: International Medical Journal of Experimental and Clinical Research, 16, BR61–BR67. 20110911

[acel13375-bib-0002] Binukumar, B. K. , Bal, A. , & Gill, K. D. (2011). Chronic dichlorvos exposure: Microglial activation, proinflammatory cytokines and damage to nigrostriatal dopaminergic system. Neuromolecular Medicine., 13, 251–265. 10.1007/s12017-011-8156-8 21964614

[acel13375-bib-0003] Cecon, E. , Oishi, A. , & Jockers, R. (2018). Melatonin receptors: Molecular pharmacology and signalling in the context of system bias. British Journal of Pharmacology, 175, 3263–3280. 10.1111/bph.13950 28707298 PMC6057902

[acel13375-bib-0004] Colonna, M. , & Butovsky, O. (2017). Microglia function in the central nervous system during health and neurodegeneration. Annual Review of Immunology, 35, 441–468. 10.1146/annurev-immunol-051116-052358 PMC8167938 28226226

[acel13375-bib-0005] Dan, L. , Wang, C. , Ma, P. , Yu, Q. , Gu, M. , Dong, L. , Jiang, W. , Pan, S. , Xie, C. , Han, J. , Lan, Y. , Sun, J. , Sheng, P. , Liu, K. , Wu, Y. , Liu, L. , Ma, Y. , & Jiang, H. (2018). PGC1alpha promotes cholangiocarcinoma metastasis by upregulating PDHA1 and MPC1 expression to reverse the Warburg effect. Cell Death & Disease., 9, 466. 10.1038/s41419-018-0494-0 29700317 PMC5919932

[acel13375-bib-0006] De Virgilio, A. , Greco, A. , Fabbrini, G. , Inghilleri, M. , Rizzo, M. I. , Gallo, A. , Conte, M. , Rosato, C. , Ciniglio Appiani, M. , & de Vincentiis, M. (2016). Parkinson's disease: Autoimmunity and neuroinflammation. Autoimmunity Reviews, 15, 1005–1011. 10.1016/j.autrev.2016.07.022 27497913

[acel13375-bib-0007] Gimeno‐Bayon, J. , Lopez‐Lopez, A. , Rodriguez, M. J. , & Mahy, N. (2014). Glucose pathways adaptation supports acquisition of activated microglia phenotype. Journal of Neuroscience Research, 92, 723–731. 10.1002/jnr.23356. 24510633

[acel13375-bib-0008] Glass, C. K. , Saijo, K. , Winner, B. , Marchetto, M. C. , & Gage, F. H. (2010). Mechanisms underlying inflammation in neurodegeneration. Cell, 140, 918–934. 10.1016/j.cell.2010.02.016 20303880 PMC2873093

[acel13375-bib-0009] Gu, C. , Hu, Q. , Wu, J. , Mu, C. , Ren, H. , Liu, C. F. , & Wang, G. (2018). P7C3 inhibits LPS‐induced microglial activation to protect dopaminergic neurons against inflammatory factor‐induced cell death in vitro and in vivo. Frontiers in Cellular Neuroscience, 12, 400. 10.3389/fncel.2018.00400 30455635 PMC6230654

[acel13375-bib-0010] Hardeland, R. , Cardinali, D. P. , Srinivasan, V. , Spence, D. W. , Brown, G. M. , & Pandi‐Perumal, S. R. (2011). Melatonin – A pleiotropic, orchestrating regulator molecule. Progress in Neurobiology, 93, 350–384. 10.1016/j.pneurobio.2010.12.004 21193011

[acel13375-bib-0011] Hefendehl, J. K. , Neher, J. J. , Suhs, R. B. , Kohsaka, S. , Skodras, A. , & Jucker, M. (2014). Homeostatic and injury‐induced microglia behavior in the aging brain. Aging Cell, 13, 60–69. 10.1111/acel.12149 23953759 PMC4326865

[acel13375-bib-0012] Hirsch, E. C. , & Hunot, S. (2009). Neuroinflammation in Parkinson’s disease: A target for neuroprotection? The Lancet Neurology, 8, 382–397. 10.1016/S1474-4422(09)70062-6 19296921

[acel13375-bib-0013] Kato, K. , Hirai, K. , Nishiyama, K. , Uchikawa, O. , Fukatsu, K. , Ohkawa, S. , Kawamata, Y. , Hinuma, S. , & Miyamoto, M. (2005). Neurochemical properties of ramelteon (TAK‐375), a selective MT1/MT2 receptor agonist. Neuropharmacology, 48, 301–310. 10.1016/j.neuropharm.2004.09.007 15695169

[acel13375-bib-0014] Kelly, B. , & O'Neill, L. A. (2015). Metabolic reprogramming in macrophages and dendritic cells in innate immunity. Cell Research, 25, 771–784. 10.1038/cr.2015.68 26045163 PMC4493277

[acel13375-bib-0015] Kuriyama, A. , Honda, M. , & Hayashino, Y. (2014). Ramelteon for the treatment of insomnia in adults: A systematic review and meta‐analysis. Sleep Medicine, 15, 385–392. 10.1016/j.sleep.2013.11.788 24656909

[acel13375-bib-0016] Lacoste, B. , Angeloni, D. , Dominguez‐Lopez, S. , Calderoni, S. , Mauro, A. , Fraschini, F. , Descarries, L. , & Gobbi, G. (2015). Anatomical and cellular localization of melatonin MT1 and MT2 receptors in the adult rat brain. Journal of Pineal Research, 58, 397–417. 10.1111/jpi.12224 25726952

[acel13375-bib-0017] Li, Y. , Lu, B. , Sheng, L. , Zhu, Z. , Sun, H. , Zhou, Y. , Yang, Y. , Xue, D. , Chen, W. , Tian, X. , Du, Y. , Yan, M. , Zhu, W. , Xing, F. , Li, K. , Lin, S. , Qiu, P. , Su, X. , Huang, Y. , … Yin, W. (2018). Hexokinase 2‐dependent hyperglycolysis driving microglial activation contributes to ischemic brain injury. Journal of Neurochemistry, 144, 186–200. 10.1111/jnc.14267 29205357

[acel13375-bib-0018] Liu, J. , Clough, S. J. , Hutchinson, A. J. , Adamah‐Biassi, E. B. , Popovska‐Gorevski, M. , & Dubocovich, M. L. (2016). MT1 and MT2 melatonin receptors: A therapeutic perspective. Annual Review of Pharmacology and Toxicology, 56, 361–383. 10.1146/annurev-pharmtox-010814-124742 PMC5091650 26514204

[acel13375-bib-0019] Miki, Y. , Tanji, K. , Mori, F. , Kakita, A. , Takahashi, H. , & Wakabayashi, K. (2017). Alteration of mitochondrial protein PDHA1 in Lewy body disease and PARK14. Biochemical and Biophysical Research Communications, 489, 439–444. 10.1016/j.bbrc.2017.05.162 28564592

[acel13375-bib-0020] Minton, K. (2017). Immunometabolism: Stress‐induced macrophage polarization. Nature Reviews. Immunology, 17, 277. 10.1038/nri.2017.41 28393924

[acel13375-bib-0021] Nair, S. , Sobotka, K. S. , Joshi, P. , Gressens, P. , Fleiss, B. , Thornton, C. , Mallard, C. , & Hagberg, H. (2019). Lipopolysaccharide‐induced alteration of mitochondrial morphology induces a metabolic shift in microglia modulating the inflammatory response in vitro and in vivo. Glia, 67, 1047–1061. 10.1002/glia.23587 30637805

[acel13375-bib-0022] Ng, K. Y. , Leong, M. K. , Liang, H. , & Paxinos, G. (2017). Melatonin receptors: Distribution in mammalian brain and their respective putative functions. Brain Structure & Function, 222, 2921–2939. 10.1007/s00429-017-1439-6 28478550

[acel13375-bib-0023] Nikbakht, F. , Belali, R. , Rasoolijazi, H. , & Mohammad Khanizadeh, A. (2019). 2‐Deoxyglucose protects hippocampal neurons against kainate‐induced temporal lobe epilepsy by modulating monocyte‐derived macrophages (mo‐MPhi) and progranulin production in the hippocampus. Neuropeptides, 76, 101932. 10.1016/j.npep.2019.05.003 31227312

[acel13375-bib-0024] O'Neill, L. A. (2014). Glycolytic reprogramming by TLRs in dendritic cells. Nature Immunology, 15, 314–315. 10.1038/ni.2852. 24646590

[acel13375-bib-0025] Orihuela, R. , McPherson, C. A. , & Harry, G. J. (2016). Microglial M1/M2 polarization and metabolic states. British Journal of Pharmacology, 173, 649–665. 10.1111/bph.13139. 25800044 PMC4742299

[acel13375-bib-0026] Peschke, E. , & Muhlbauer, E. (2010). New evidence for a role of melatonin in glucose regulation. Best Practice & Research Clinical Endocrinology & Metabolism, 24, 829–841. 10.1016/j.beem.2010.09.001 21112029

[acel13375-bib-0027] Reiter, R. J. , Calvo, J. R. , Karbownik, M. , Qi, W. , & Tan, D. X. (2000). Melatonin and its relation to the immune system and inflammation. Annals of the New York Academy of Sciences, 917, 376–386 11268363 10.1111/j.1749-6632.2000.tb05402.x

[acel13375-bib-0028] Roth, T. , Stubbs, C. , & Walsh, J. K. (2005). Ramelteon (TAK‐375), a selective MT1/MT2‐receptor agonist, reduces latency to persistent sleep in a model of transient insomnia related to a novel sleep environment. Sleep, 28, 303–307. 16173650

[acel13375-bib-0029] Sanchez, A. , Calpena, A. C. , & Clares, B. (2015). Evaluating the oxidative stress in inflammation: Role of melatonin. International Journal of Molecular Sciences, 16, 16981–17004. 10.3390/ijms160816981 26225957 PMC4581180

[acel13375-bib-0030] Semba, H. , Takeda, N. , Isagawa, T. , Sugiura, Y. , Honda, K. , Wake, M. , Miyazawa, H. , Yamaguchi, Y. , Miura, M. , Jenkins, D. M. , Choi, H. , Kim, J. W. , Asagiri, M. , Cowburn, A. S. , Abe, H. , Soma, K. , Koyama, K. , Katoh, M. , Sayama, K. , … Komuro, I. (2016). HIF‐1alpha‐PDK1 axis‐induced active glycolysis plays an essential role in macrophage migratory capacity. Nature Communications, 7, 11635. 10.1038/ncomms11635 PMC4873978 27189088

[acel13375-bib-0031] Stauch, B. , Johansson, L. C. , McCorvy, J. D. , Patel, N. , Han, G. W. , Huang, X. P. , Gati, C. , Batyuk, A. , Slocum, S. T. , Ishchenko, A. , Brehm, W. , White, T. A. , Michaelian, N. , Madsen, C. , Zhu, L. , Grant, T. D. , Grandner, J. M. , Shiriaeva, A. , Olsen, R. H. J. , … Cherezov, V. (2019). Structural basis of ligand recognition at the human MT1 melatonin receptor. Nature, 569, 284–288. 10.1038/s41586-019-1141-3 31019306 PMC6696938

[acel13375-bib-0032] Sulkava, S. , Muggalla, P. , Sulkava, R. , Ollila, H. M. , Peuralinna, T. , Myllykangas, L. , Kaivola, K. , Stone, D. J. , Traynor, B. J. , Renton, A. E. , Rivera, A. M. , Helisalmi, S. , Soininen, H. , Polvikoski, T. , Hiltunen, M. , Tienari, P. J. , Huttunen, H. J. , & Paunio, T. (2018). Melatonin receptor type 1A gene linked to Alzheimer's disease in old age. Sleep, 41, zsy103. 10.1093/sleep/zsy103 PMC6047434 29982836

[acel13375-bib-0033] Suofu, Y. , Li, W. , Jean‐Alphonse, F. G. , Jia, J. , Khattar, N. K. , Li, J. , Baranov, S. V. , Leronni, D. , Mihalik, A. C. , He, Y. , Cecon, E. , Wehbi, V. L. , Kim, J. , Heath, B. E. , Baranova, O. V. , Wang, X. , Gable, M. J. , Kretz, E. S. , Di Benedetto, G. , … Friedlander, R. M. (2017). Dual role of mitochondria in producing melatonin and driving GPCR signaling to block cytochrome c release. Proceedings of the National Academy of Sciences of the United States of America, 114, E7997–E8006. 10.1073/pnas.1705768114 28874589 PMC5617277

[acel13375-bib-0034] Tan, Z. , Xie, N. , Cui, H. , Moellering, D. R. , Abraham, E. , Thannickal, V. J. , & Liu, G. (2015). Pyruvate dehydrogenase kinase 1 participates in macrophage polarization via regulating glucose metabolism. Journal of Immunology, 194, 6082–6089. 10.4049/jimmunol.1402469 PMC4458459 25964487

[acel13375-bib-0035] Van den Bossche, J. , O'Neill, L. A. , & Menon, D. (2017). Macrophage immunometabolism: Where are we (going)? Trends in Immunology, 38, 395–406. 10.1016/j.it.2017.03.001 28396078

[acel13375-bib-0036] Wang, X. , Sirianni, A. , Pei, Z. , Cormier, K. , Smith, K. , Jiang, J. , Zhou, S. , Wang, H. , Zhao, R. , Yano, H. , Kim, J. E. , Li, W. , Kristal, B. S. , Ferrante, R. J. , & Friedlander, R. M. (2011). The melatonin MT1 receptor axis modulates mutant Huntingtin‐mediated toxicity. The Journal of Neuroscience: The Official Journal of the Society for Neuroscience, 31, 14496–14507. 10.1523/JNEUROSCI.3059-11.2011 21994366 PMC3213696

[acel13375-bib-0037] Wolf, S. A. , Boddeke, H. W. , & Kettenmann, H. (2017). Microglia in physiology and disease. Annual Review of Physiology, 79, 619–643. 10.1146/annurev-physiol-022516-034406 27959620

[acel13375-bib-0038] Wu, Y. H. , Zhou, J. N. , Van Heerikhuize, J. , Jockers, R. , & Swaab, D. F. (2007). Decreased MT1 melatonin receptor expression in the suprachiasmatic nucleus in aging and Alzheimer's disease. Neurobiology of Aging, 28, 1239–1247. 10.1016/j.neurobiolaging.2006.06.002. 16837102

[acel13375-bib-0039] Yu, Y. X. , Li, Y. P. , Gao, F. , Hu, Q. S. , Zhang, Y. , Chen, D. , & Wang, G. H. (2016). Vitamin K2 suppresses rotenone‐induced microglial activation in vitro. Acta Pharmacologica Sinica, 37, 1178–1189. 10.1038/aps.2016.68 27498777 PMC5022102

[acel13375-bib-0040] Zlotos, D. P. , Jockers, R. , Cecon, E. , Rivara, S. , & Witt‐Enderby, P. A. (2014). MT1 and MT2 melatonin receptors: Ligands, models, oligomers, and therapeutic potential. Journal of Medicinal Chemistry, 57, 3161–3185. 10.1021/jm401343c. 24228714

