# Correction to: Microglial MT1 activation inhibits LPS‐induced neuroinflammation via regulation of metabolic reprogramming

**DOI:** 10.1111/acel.14068

**Published:** 2023-12-20

Gu, C., Wang, F., Zhang, Y.‐T., Wei, S.‐Z., Liu, J.‐Y., Sun, H.‐Y., Wang, G.‐H., & Liu, C.‐F. (2021). Microglial MT1 activation inhibits LPS‐induced neuroinflammation via regulation of metabolic reprogramming. *Aging Cell*, 20(6), e13375. https://doi.org/10.1111/acel.13375

Two figures (Figure 1 and Figure 6e) were incorrectly presented.

In Figure 1, we accidentally put the cell morphology of primary microglia in other fields of view in the Ramelteon group in Figure 1n into the si‐Control group in Figure 1g. However, this does not affect the conclusion of the article.

In Figure 6e, the labeling of statistical graphs of IBA1 protein level and TH protein level have been reversed.

The correct figures are shown below.

**Figure:**
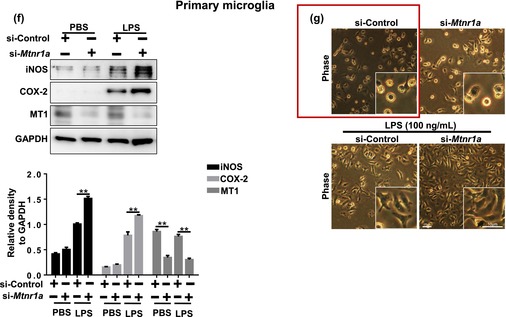
FIGURE 1

**Figure:**
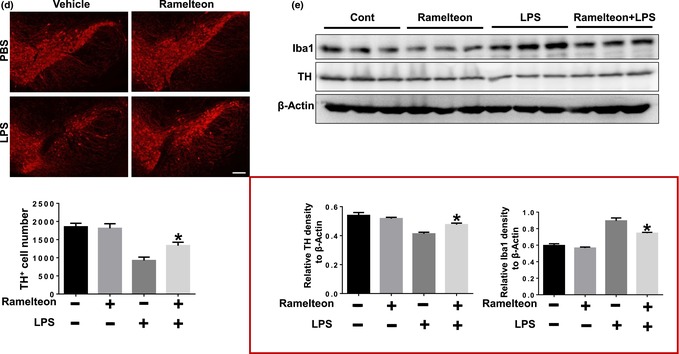
FIGURE 6

We apologize for this error.

