# Supplementary material for: Microglial MT1 activation inhibits LPS‐induced neuroinflammation via regulation of metabolic reprogramming
Source: Aging Cell. 2021 May 8;20(6):e13375. doi: 10.1111/acel.13375 (PMC8208780; doi:10.1111/acel.13375)
Supplement: Supplementary file 1 — Supplementary Material [file ACEL-20-e13375-s001.docx]

**Microglial MT1 activation inhibits LPS-induced neuroinflammation via regulation of metabolic reprogramming**

***Supplementary information***

**
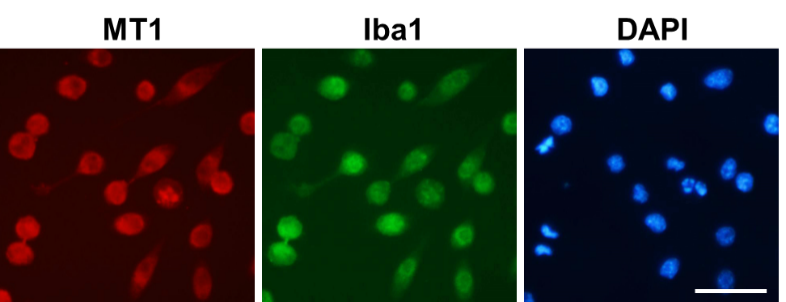

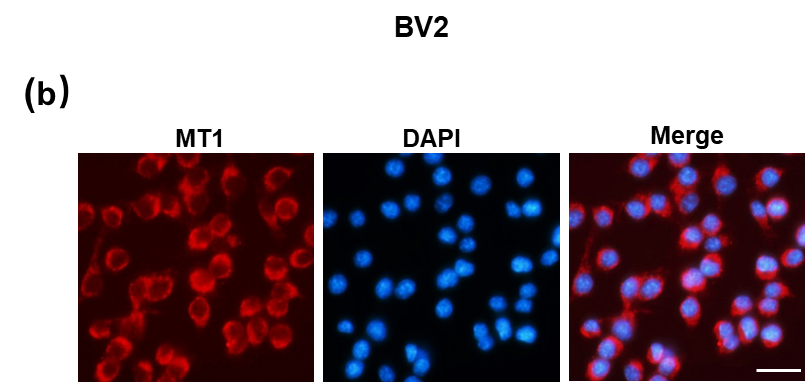
(a)**

**Supplementary Figure 1. MT1 is expressed in primary microglia and BV2 microglial cells.** (a) The expression of MT1 (green) and Iba1 (red) in primary microglia. Scale bar, 20 μm. (b) The expression of MT1 (red) in microglial BV2 cells. Scale bar, 20 μm.


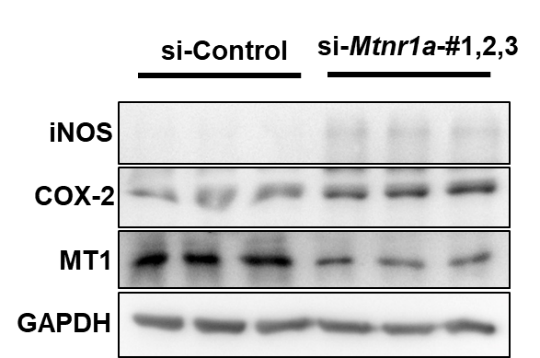


**Supplementary Figure 2. The knockdown efficiency of the siRNAs against *Mtnr1a*.** BV2 cells were transfected with control siRNA or *Mtnr1a* siRNA for 48 h, After treatments, the protein levels of iNOS, COX-2, MT1 and GAPDH were determined using immunoblot analyses.


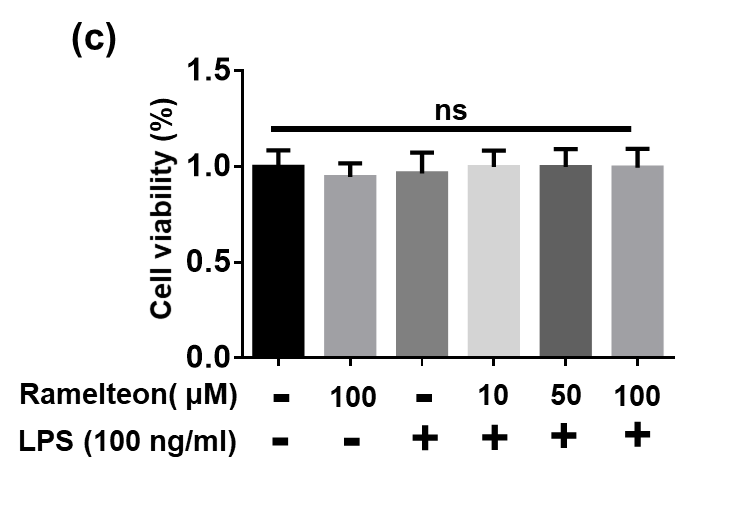

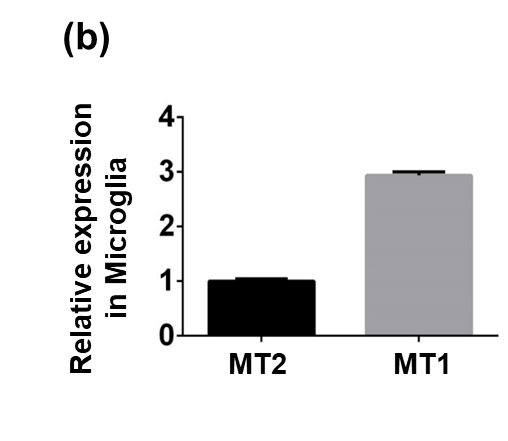

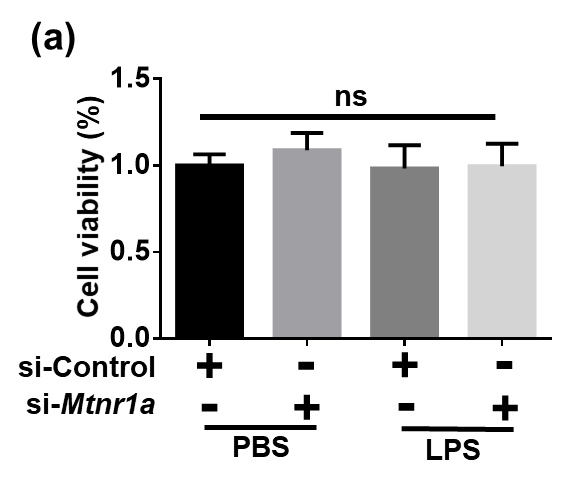


**Supplementary Figure 3.** **Cell viability analysis after si-*Mtnr1a* transfection or Ramelteon treatment.** (a) BV2 cells were transfected with control siRNA or *Mtnr1a* siRNA for 48 h, then exposed to LPS (100 ng/mL) for 12 h. After treatments, the cell viability was detected with MTT assays. (b) The mRNA levels of MT1 and MT2 in microglia were detected using qRT-PCR assays. (c) BV2 cells were pretreated with Ramelteon (10 μM, 50 μM, 100 μM) for 12 h and then exposed to LPS (100 ng/mL) for 12 h. Thereafter, the cell viability was detected with MTT assays. ns, no significance, two-way ANOVA followed by Sidak’s post-hoc test.


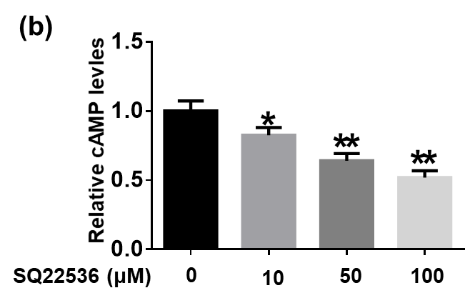

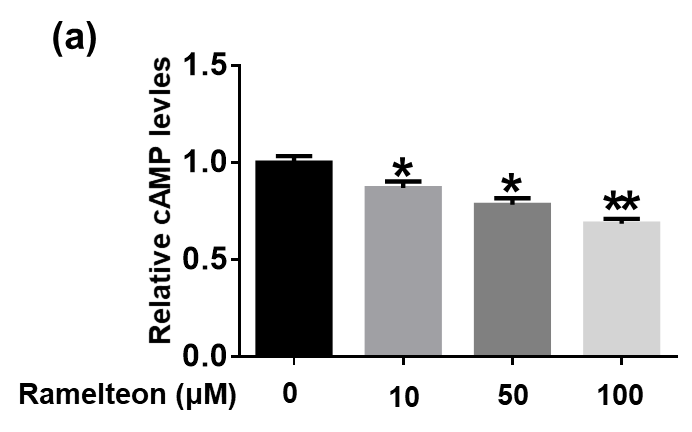


**Supplementary Figure 4. The cAMP levels in BV2 after Ramelteon or SQ22536 treatment. (a)** BV2 cells were treated with Ramelteon (10 μM, 50 μM, 100 μM) for 12 h, then the cAMP levels in BV2 was assessed. **P* < 0.05, ***P* < 0.01 vs. the group without Ramelteon treatment, one-way ANOVA followed by t test. (b) BV2 cells were treated with SQ22536 (10 μM, 50 μM, 100 μM) for 12 h, followed by the detection of cAMP levels. **P* < 0.05, ***P* < 0.01 vs. the group without Ramelteon treatment, one-way ANOVA followed by t test.

**
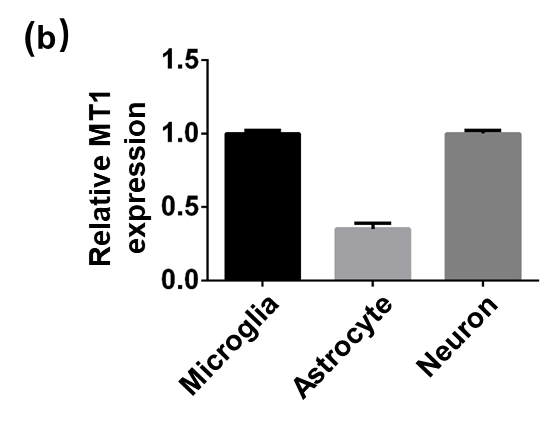
(a)**


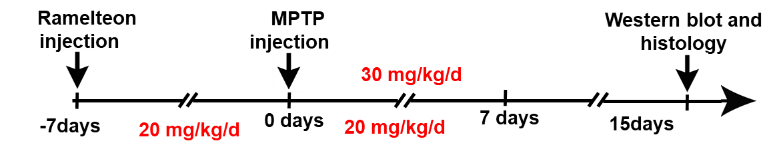


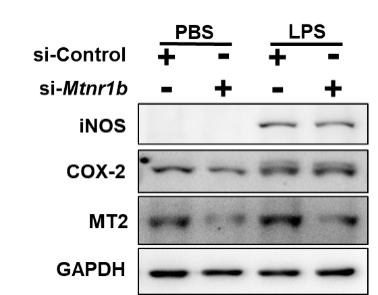


**(c)**

**Supplementary Figure 5.** (a) A schematic diagram showed the animal experimental procedure in MPTP-induced PD mouse model. Before MPTP treatment, a seven consecutive days intraperitoneal injections of Ramelteon were conducted, then MPTP intraperitoneal injections along with Ramelteon treatment were performed for another seven consecutive days, then western blot and histology were detected after 7 days. (b) The mRNA levels of MT1 in cultured primary microglia, astrocyte and neuron were detected using qRT-PCR assays *in vitro*. (c) MT2 deficiency had no influence on LPS-induced production of pro-inﬂammatory factors in microglia.

**
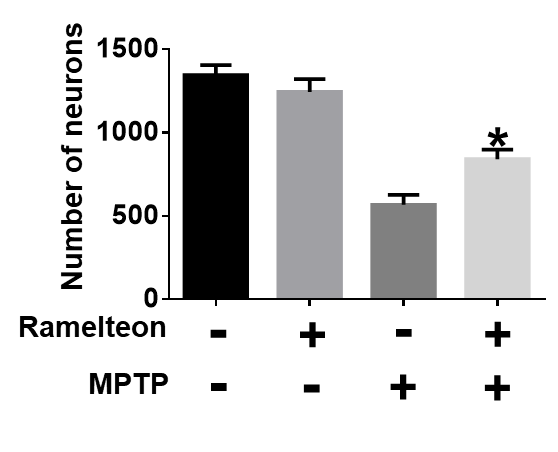

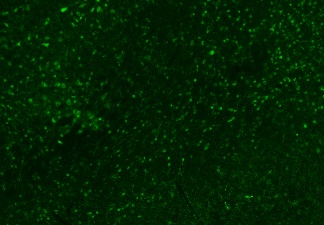

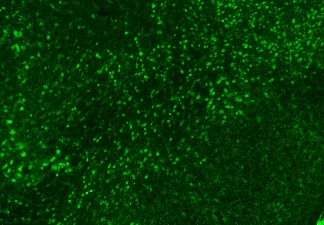

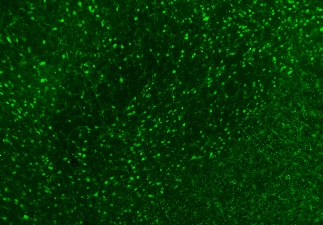

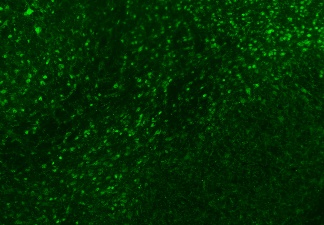
**

**(a)**

**Ramelteon**

**PBS**

**MPTP**

**Vehicle**

**(b)**

**
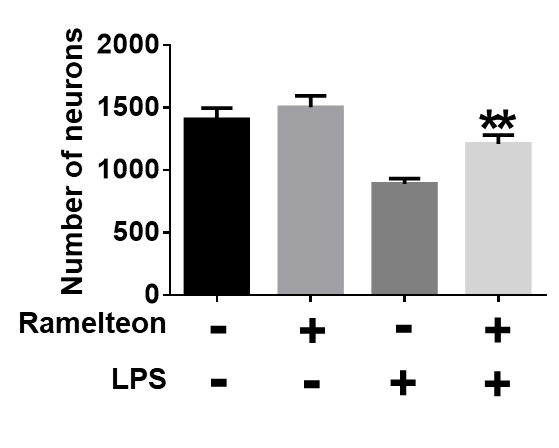
**
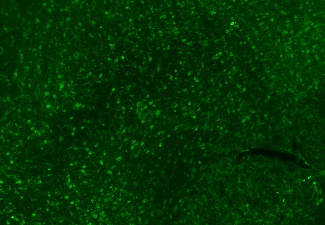

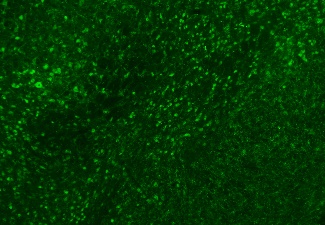
**
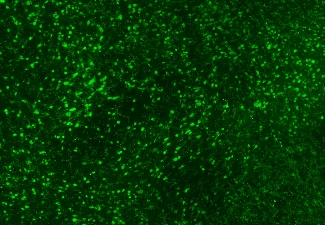

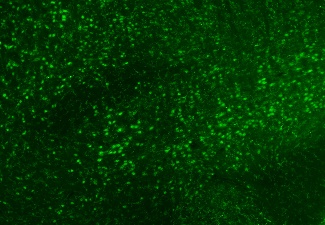
**

**LPS**

**PBS**

**Ramelteon**

**Vehicle**


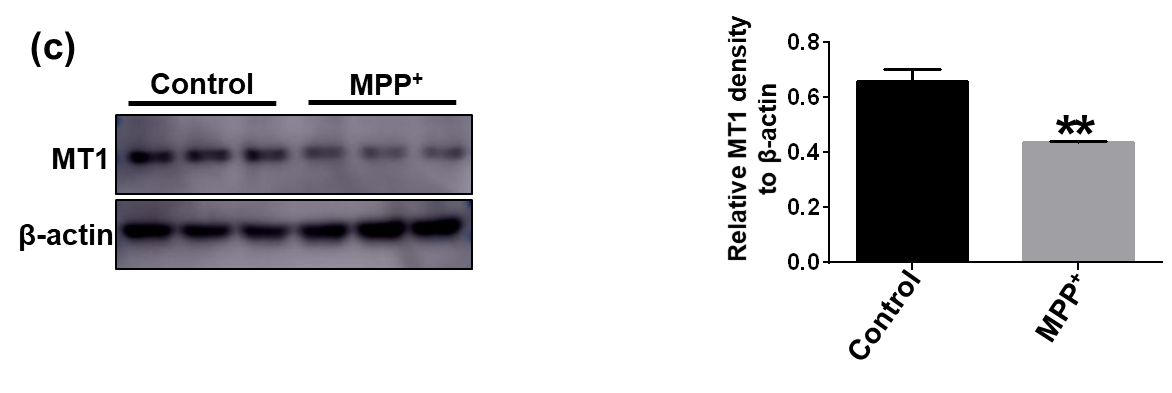


**Supplementary Figure 6**. (a) Immunohistochemical staining was performed via anti-NeuN antibody. The quantification of neuronal cell number in SN was shown in the right panel. Scale bar, 50 μm. n = 5 per group. **P* < 0.01 vs. the group treated with MPTP alone using two-way ANOVA followed by Sidak’s post-hoc test. (b) Immunohistochemical staining was conducted using anti-NeuN antibody. The quantification of neuronal cell number in SN was shown in the right panel. Scale bar, 50 μm. n = 4 per group. ***P* < 0.01 vs. the group treated with LPS alone, two-way ANOVA followed by Sidak’s post-hoc test. (c) Primary microglia were treated with MPP^+^ (250 μM) for 12 h. Then the total protein were collected for measure the levels of MT1 in MPP^+^-treated group using immunoblot analyses. Quantitative analyses were shown in the right panel. n = 3 per group. ***P* < 0.01 vs. the group treated with MPP^+^ alone, one-way ANOVA followed by t test.
